# Improving Infant Hydrocephalus Outcomes in Uganda: A Longitudinal Prospective Study Protocol for Predicting Developmental Outcomes and Identifying Patients at Risk for Early Treatment Failure after ETV/CPC

**DOI:** 10.3390/metabo12010078

**Published:** 2022-01-14

**Authors:** Taylor A. Vadset, Ajay Rajaram, Chuan-Heng Hsiao, Miriah Kemigisha Katungi, Joshua Magombe, Marvin Seruwu, Brian Kaaya Nsubuga, Rutvi Vyas, Julia Tatz, Katharine Playter, Esther Nalule, Davis Natukwatsa, Moses Wabukoma, Luis E. Neri Perez, Ronald Mulondo, Jennifer T. Queally, Aaron Fenster, Abhaya V. Kulkarni, Steven J. Schiff, Patricia Ellen Grant, Edith Mbabazi Kabachelor, Benjamin C. Warf, Jason D. B. Sutin, Pei-Yi Lin

**Affiliations:** 1 Division of Newborn Medicine, Boston Children’s Hospital, Boston, MA 02115, USA; taylor.vadset@childrens.harvard.edu (T.A.V.); ajay.rajaram@childrens.harvard.edu (A.R.); chuan-heng.hsiao@childrens.harvard.edu (C.-H.H.); rutvi.vyas@childrens.harvard.edu (R.V.); julia.tatz@childrens.harvard.edu (J.T.); katplayter@gmail.com (K.P.); Luis.NeriPerez@childrens.harvard.edu (L.E.N.P.); ellen.grant@childrens.harvard.edu (P.E.G.); jason.sutin@childrens.harvard.edu (J.D.B.S.); 2 Fetal-Neonatal Neuroimaging and Developmental Science Center, Boston Children’s Hospital, Boston, MA 02115, USA; 3 Department of Pediatrics, Harvard Medical School, Boston, MA 02115, USA; 4 CURE Children’s Hospital of Uganda, Mbale P.O. Box 903, Uganda; miriah.kemigisha@cureinternational.org (M.K.K.); joshua.magombe@cureinternational.org (J.M.); marvin.seruwu@cureinternational.org (M.S.); brian.nsubuga.kaaya@cure.org (B.K.N.); esther.nalule@cureinternational.org (E.N.); davis.natukwatsa@cure.org (D.N.); moses.wabukoma@cure.org (M.W.); mulondor2@gmail.com (R.M.); edith.mbabazi@cureinternational.org (E.M.K.); 5 Department of Psychiatry, Boston Children’s Hospital, Harvard Medical School, Boston, MA 02115, USA; jennifer.queally@childrens.harvard.edu; 6 Robarts Research Institute, Western University, London, ON N6A 3K7, Canada; afenster@robarts.ca; 7 The Hospital for Sick Children, Toronto, ON M5G 1X8, Canada; abhaya.kulkarni@sickkids.ca; 8 Center for Neural Engineering, Center for Infectious Disease Dynamics, Departments of Engineering Science and Mechanics, Neurosurgery, and Physics, The Pennsylvania State University, University Park, PA 16802, USA; steven.schiff@psu.edu; 9 Department of Radiology, Boston Children’s Hospital, Harvard Medical School, Boston, MA 02115, USA; 10 Department of Neurosurgery, Boston Children’s Hospital, Harvard Medical School, Boston, MA 02115, USA; benjamin.warf@childrens.harvard.edu

**Keywords:** hydrocephalus, cerebral oxygen metabolism, cerebral blood flow, tissue saturation, brain growth, neurodevelopmental outcome, frequency-domain near-infrared spectroscopy, diffuse correlation spectroscopy, endoscopic third ventriculostomy combined with choroid plexus cauterization

## Abstract

Infant hydrocephalus poses a severe global health burden; 80% of cases occur in the developing world where patients have limited access to neurosurgical care. Surgical treatment combining endoscopic third ventriculostomy and choroid plexus cauterization (ETV/CPC), first practiced at CURE Children’s Hospital of Uganda (CCHU), is as effective as standard ventriculoperitoneal shunt (VPS) placement while requiring fewer resources and less post-operative care. Although treatment focuses on controlling ventricle size, this has little association with treatment failure or long-term outcome. This study aims to monitor the progression of hydrocephalus and treatment response, and investigate the association between cerebral physiology, brain growth, and neurodevelopmental outcomes following surgery. We will enroll 300 infants admitted to CCHU for treatment. All patients will receive pre/post-operative measurements of cerebral tissue oxygenation (SO_2_), cerebral blood flow (CBF), and cerebral metabolic rate of oxygen consumption (CMRO_2_) using frequency-domain near-infrared combined with diffuse correlation spectroscopies (FDNIRS-DCS). Infants will also receive brain imaging, to monitor tissue/ventricle volume, and neurodevelopmental assessments until two years of age. This study will provide a foundation for implementing cerebral physiological monitoring to establish evidence-based guidelines for hydrocephalus treatment. This paper outlines the protocol, clinical workflow, data management, and analysis plan of this international, multi-center trial.

## 1. Introduction

Hydrocephalus is a severe disease where excess cerebral spinal fluid (CSF) accumulates in the brain, and if left untreated will rapidly cause brain damage, severe neurological and developmental disorders, and even death. While the global health burden of infant hydrocephalus is substantial for rich and poor countries alike, it is especially prominent in sub-Saharan Africa (SSA), where approximately 180,000 new cases arise each year [1,2]. In low- and middle-income countries (LMICs), post-infectious hydrocephalus (PIH) following neonatal sepsis is the most common form of infant hydrocephalus [3]. Regardless of etiology, prompt surgical intervention prevents dire outcomes and leads to more favorable results for these children [4,5].

Traditionally, infants with hydrocephalus are treated with a ventriculoperitoneal shunt (VPS), a permanently-implanted mechanical valve that drains CSF directly from the brain’s ventricles into the abdominal cavity to ease the enlargement of the ventricles [6,7]. However, VPS is prone to frequent failures from mechanical obstructions, which requires urgent shunt revision surgery [8]. In LMICs where immediate neurosurgical care is limited, shunt failure can be fatal [1,3,4]. The CURE Children’s Hospital of Uganda (CCHU) innovated the treatment of infant hydrocephalus by developing the implant-free endoscopic third ventriculostomy and choroid plexus cauterization (ETV/CPC) procedure, which diverts the flow of CSF (through ETV) and reduces CSF production (through CPC) [9,10,11,12,13,14,15]. In comparison to shunt placement, successful ETV/CPC treatment lowers infection risk and presents a similar mortality or morbidity rate. More importantly, ETV/CPC avoids life-long shunt dependence with its attendant danger of shunt failure, has a lower risk of treatment failure after six months, and may ultimately be more cost-effective [9,10,11,12,13,14,15]. The randomized controlled trial (RCT) at CCHU comparing ETV/CPC vs. VPS treatments (NCT01936272) found no difference in effectiveness or neurodevelopmental outcomes at one and two years of follow-up [16,17]. Additionally, a multi-center RCT (NCT04177914) assessing the efficacy of ETV/CPC vs. VPS in hydrocephalus patients with etiologies other than PIH is also ongoing in North America.

Despite the known benefits of successful ETV/CPC treatment for infant hydrocephalus, treatment failure remains a significant problem for about one-third of cases [1,3,18], most of which occur within six months with little occurring thereafter [18]. In early ETV/CPC failures, the ETV is typically open and, often, no explanation for the cause of failure is evident within structural imaging [19]. Few risk factors for early failure have been identified, and those that have are mostly related to patient demographics such as age at time of treatment [10]. Therefore, diagnostics to detect early treatment failure, evaluate treatment response and likelihood of future failure, and stratify pre-treatment patients are urgent unmet medical needs for improving infant hydrocephalus outcomes.

The goal of hydrocephalus treatment has traditionally been to ease the progressive enlargement of ventricle size. Thus, diagnosis and monitoring of hydrocephalus largely relies on brain imaging tools such as head ultrasound (HUS), computed tomography (CT), and magnetic resonance imaging (MRI). Physiological-based diagnostics for hydrocephalus are attractive alternatives to those based on brain structure because such measures are more closely related to the pathophysiology of the disease. Near-infrared spectroscopy (NIRS) measures cerebral oxygen saturation (SO_2_) and is a widely available means of physiological monitoring that can be employed safely and conveniently at the bedside. Although SO_2_ is easy to measure, it does not always offer a comprehensive understanding of infant brain pathophysiology and disease progression [20], and has limited diagnostic value for hydrocephalus [21].

We have developed novel frequency-domain near-infrared combined with diffuse correlation spectroscopy (FDNIRS-DCS) technology to measure SO_2_, cerebral blood flow (CBF), and cerebral metabolic rate of oxygen consumption (CMRO_2_) non-invasively. We have demonstrated that these measurements are feasible at the bedside [22,23] and sensitive in measuring common infant brain pathologies, including intraventricular hemorrhage [24] and hypoxic-ischemic encephalopathy [25]. In pilot studies at Boston Children’s Hospital (BCH) and CCHU, we show they are also sensitive to monitoring the progression and treatment of post-hemorrhagic hydrocephalus (PHH) and PIH. Additionally, in part by the results of the recent RCT (NCT01936272) at CCHU, which found brain growth after treatment is associated with long-term outcomes [16,17], we hypothesize increased brain metabolism to be a precursor to brain growth. Its measurement has potential as a new biomarker for hydrocephalus, which may provide early, quantitative indications of disease state before its effects manifest in the growth rate.

In this study, we propose the first large-scale clinical study to monitor the longitudinal evolution of cerebral physiology (SO_2_, CBF, and CMRO_2_), brain volume growth, and neurodevelopmental outcomes up to two years of age in hydrocephalus infants treated at CCHU. We will investigate associations of CBF and CMRO_2_ with long-term brain growth and neurodevelopmental outcomes, and also explore the utility of FDNIRS-DCS measures as new biomarkers to aid the prediction of treatment failure. Success in our study goals will open a new avenue for diagnosis and treatment of hydrocephalus, provide more relevant treatment goals, and enable the search for modifiable factors to improve outcomes [25]. The aims of this study are as follows:

Aim 1: To establish the first large-scale dataset of the longitudinal evolution of cerebral physiology, structure, treatment outcome, and long-term neurodevelopmental outcome during hydrocephalus progression.
  (a) Brain physiology: Perform FDNIRS-DCS measurements during hospital stay and at each follow-up exam to determine SO_2_, CBF, and CMRO_2_.
  (b) Brain structure: Perform a CT/HUS head scan pre-operatively and post-operatively at day one, six and 12 months, and at 24 months of age to determine brain and CSF volumes as well as extra-axial layer thickness.
  (c) Clinical course: Follow response to treatment and occurrence of treatment failure.
  (d) Neurodevelopmental outcome: Following the baseline assessment, evaluate the outcomes at six and 12 months post-operatively, and at 24 months of age with the Bayley Scales of Infant and Toddler Development, Third Edition (BSID-3) and the Vineland Adaptive Behavior Scales, Third Edition (Vineland-3).
Aim 2: To determine the statistical power of optical measures for early prediction of treatment failure, neurodevelopmental outcome, and post-treatment brain growth.

## 2. Experimental Design

This protocol paper outlines the establishment of an international, multi-center investigation to address the research aims noted above. As such, this section describes the finalized experimental design and clinical/research workflow for the proposed work. Detailed information on the data collection, management, and analytical plan is discussed in Section 3.

### 2.1. Study Design

This is an investigator-initiated, multi-site, prospective, observational study that seeks to characterize the longitudinal evolution of cerebral physiology and brain growth during disease progression, treatment response, and long-term neurodevelopment up to two years of age in children with hydrocephalus. We aim to determine the utility of novel FDNIRS-DCS technology as a tool for predicting treatment failure, post-treatment brain growth, and neurodevelopmental outcome.

### 2.2. Study Organization

Boston Children’s Hospital (BCH) is the overall responsible site for this study. Research staff involved include the principal investigator (PI), co-investigators, clinical psychologist, data analyst, data manager, software engineer, and engineering and clinical research assistants. The PI and the BCH team are responsible for all technical responsibilities related to FDNIRS-DCS instrument/probe development and maintenance, the development of software interfaces and algorithms for data analysis, study staff training at CCHU for the FDNIRS-DCS data collection in infants, and the provision of long-distance technical support. In addition, the PI is also responsible for ascertaining the scientific accuracy of all published material resulting from this proposal, assuming fiscal and administrative management, keeping active communications with other key personnel and research staff at all sites, compiling and submitting the progress reports to the sponsor, managing the IRB regulatory aspects, and coordinating annual meetings. The BCH team will travel to CCHU at least twice a year to support the study’s progress.

CURE Children’s Hospital of Uganda (CCHU), located in Mbale, Uganda, is the primary recruitment site and has been a center of excellence for neurosurgery treatment, training, and research for over 20 years. CCHU is the main referral center for patients with neurosurgical conditions from all over Uganda, as well as for neighboring countries such as Kenya, Tanzania, Democratic Republic of the Congo, Rwanda, and South Sudan. On-site staff are responsible for patient recruitment and enrollment, clinical and research data acquisition and entry, and all study follow-up procedures. CCHU staff include the site-PI, study coordinator, assessments coordinator, tracking coordinator, data manager and statistician, research IT technician, study radiographer/technician, and data research assistant. The CCHU site-PI is responsible for overseeing all day-to-day aspects of the project as they relate to the Uganda site, as well as the CCHU personnel, and ensures the project meets its stated aims and stays on schedule from screening and enrollment through published results.

There are several other participating sites involved in data acquisition and analysis. The Pennsylvania State University (PSU) oversees the design and development of software for brain and CSF volumetric analysis from CT imaging. Western University (WU), in London, Canada, oversees the hardware and software development for volumetric HUS imaging. The Hospital for Sick Children, in Toronto, Canada, is responsible for analyzing the data collected from the neurodevelopmental assessments.

### 2.3. Study Period

Patient enrollment began in May 2021 and will continue through January 2023. All enrolled patients will be followed until they are 24 months of age or until death. If informed consent is withdrawn by the patient’s primary caregiver, all prospective research activity relating to the infant will be immediately halted.

### 2.4. Participants

We plan to enroll 300 infants who have progressive symptomatic hydrocephalus or are at risk for developing hydrocephalus due to a spina bifida or encephalocele diagnosis and are considered eligible for the ETV/CPC procedure. They will be recruited over a period of 20 months at CCHU. The recruitment period is estimated based on the randomized control clinical trial (NCT01936272) previously conducted at CCHU. Inclusion criteria are: (1) patients who are less than six months of age; (2) live in the Northern, Central, or Eastern districts of Uganda; (3) have a parent or guardian qualified by Ugandan law to give informed consent. Patients are excluded if they fail to meet the inclusion criteria or have a history of endoscopic third ventriculostomy or ventriculoperitoneal shunt placement to treat hydrocephalus prior to consent. No subject will be excluded based on gender, race, and/or socioeconomic status.

### 2.5. Patient Screening and Recruitment

CCHU admits approximately 100 infants with hydrocephalus each month. Some of these patients are referred by outside clinics, but the majority are self-referred. All patients admitted through the outpatient department will be screened for eligibility by the site-PI or the study coordinator. If deemed eligible for the study, CCHU staff will consent and enroll the patient into the study.

### 2.6. Informed Consent

The parent/guardian is asked to decide whether or not to participate in the study after being fully informed of the nature of the study and how it will and will not affect their child, as explained in the consent form. It is stressed that their child’s care will not be in any way compromised if they decide not to participate in the study or withdraw at a later time.

Consent is obtained from the parent(s)/guardian of qualifying infants shortly after the child is admitted to CCHU, identified as meeting the inclusion criteria, and after the parent(s)/guardian has been provided the opportunity to have any questions answered or concerns addressed. Although English is the official language of Uganda, the consent form has been translated into the seven most commonly spoken local languages in the Eastern, Central, and Northern regions of Uganda. For parents who are not fluent in any of these languages, consent is only obtained through an interpreter who explains the study and the consent form to them in their own language. For an illiterate parent, an identifying thumbprint is obtained in lieu of a signature in the presence of an impartial witness. This is the current culturally accepted method of obtaining operative or other medical-related consents at CCHU [16].

### 2.7. Clinical Workflow

Infants arriving through CCHU’s outpatient department are screened by a physician or healthcare worker to evaluate condition severity. Upon admission, infants receive a non-contrast head CT scan for diagnosis and surgical decision making. Prior to surgery, patients often receive a blood draw or ventricular tap to assess CSF abnormalities or infection. If a CSF culture indicates infection, surgery will be pending and the infant is sent home on antibiotics barring additional concerns. Once surgery is confirmed, the patient will be re-admitted to the hospital before the operation. Following the procedure, patients are admitted to the ICU for overnight monitoring before being transferred to the Neuro Ward. In the days following, if the patient’s post-operative course is non-problematic, the patient will be discharged. Patient follow-ups are regularly conducted at CCHU or a satellite clinic.

### 2.8. Intervention

The standard clinical protocol to treat hydrocephalus is by ETV/CPC when feasible, and this study will not impact any clinical decision making or patient care. ETV/CPC comprises a standard frontal approach with flexible endoscopy, as previously described by Warf [15]. Patients who are not eligible for ETV/CPC due to ventricular distortion or arachnoid scarring of the prepontine cistern will be treated by inserting a VPS instead. However, arachnoid scarring of the prepontine cistern that prevents ETV would only be observed during the operation, thus some enrolled patients may receive a VPS. In this study, we are enrolling subjects for whom the intended form of treatment is ETV/CPC and anticipating 20% of infants will cross to VPS [16]. The shunt insertion comprises a standard frontal approach VPS using a silastic Chhabra system, as previously described by Warf [5]. Patients will be clinically monitored for two to five days after surgery, depending on the recovery course from the operation.

The end point for this study’s treatment failure assessment is six months post-surgery, as the majority (pooled success rate of 63%, range 54–72%) of ETV failures occur before that time point [10,26]. Surgical failure is defined as the need for any subsequent surgical procedure, including ETV revision, insertion of VPS, or shunt revision for definitive CSF diversion, as well as death related to hydrocephalus management within the first six months.

### 2.9. Research Workflow

**Figure 1:**
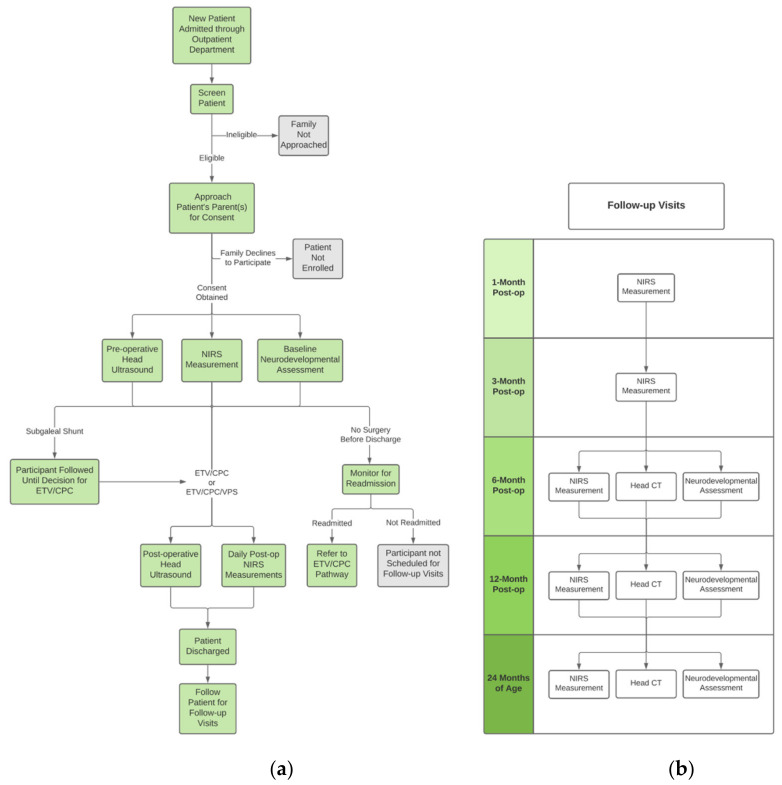
(**a**) Clinical and research workflow, (**b**) Scheduled patient research follow-ups.

The study workflow for each patient is depicted in Figure 1a. Upon enrollment, each infant undergoes a pre-operative HUS in addition to their clinically-warranted head CT scan. At least one pre-operative FDNIRS-DCS measurement is completed, depending on the duration of the participant’s pre-operative inpatient stay. A patient’s baseline neurodevelopmental assessment is also performed prior to surgery. A HUS scan is acquired post-operatively, as well as daily FDNIRS-DCS measurements for the remainder of the infant’s hospital stay.

Study follow-up visits are coordinated by staff to take place on the same day as each infant’s clinical follow-up appointment. These are completed one, three, six, and 12 months post-operatively, as well as at 24 months of age (Figure 1b). At one and three months, another FDNIRS-DCS measurement is acquired. Study procedures occurring at the six and 12 month post-op and 24 months of age time points include an FDNIRS-DCS measurement, head CT scan, and a neurodevelopmental outcome assessment.

## 3. Results

### 3.1. Data Collection

The detailed timeline for each assessment is presented in Figure 1. In addition to the scheduled visits, any acute clinical deterioration may mandate an urgent unscheduled clinical visit for further assessment. If this occurs and if approved by the infant’s clinical care team, CCHU staff will perform additional study measurements.

#### 3.1.1. Patient Demographics and Clinical Data Collection

At the time of initial treatment, baseline clinical data including birth date, region of residence, age at treatment (months), weight (kg), head circumference (cm, percentile for age), date of illness prior to onset of hydrocephalus, and date hydrocephalus first noted is collected. Prior to surgery, routine perioperative laboratory results such as complete blood counts, CSF analysis, and tests for electrolytes, malaria, and HIV, will also be recorded by CCHU staff and entered in the case report form (CRF).

#### 3.1.2. CT and US Imaging

As part of their routine clinical assessment, all hydrocephalus patients have at least one non-contrast CT scan upon admission to determine the diagnosis and for pre-operative planning. For enrolled subjects, additional head ultrasound and/or CT scans are performed at day one post-surgery, six and 12 months post-operatively, and 24 months of age. HUS measurements consist of standard clinical 2D-US views, transcranial doppler (TCD) measures of flow velocity in the anterior and middle cerebral arteries, as well as sagittal and coronal 3D-US scans acquired using proprietary hardware and software (described in Section 3.3.3) [27].

#### 3.1.3. FDNIRS-DCS Measurements

**Figure 2:**
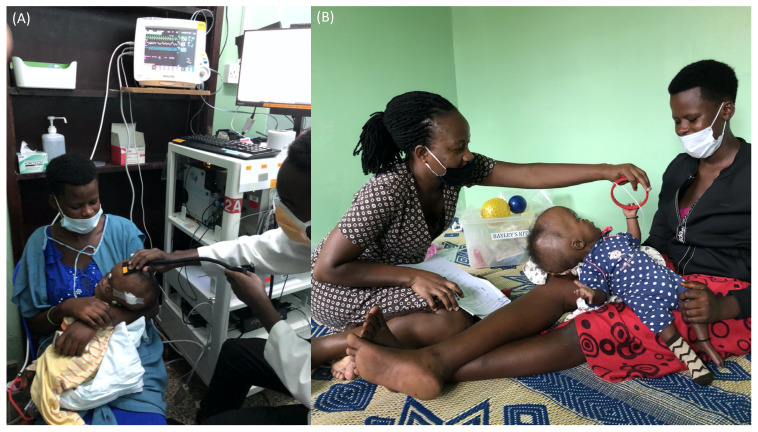
Example of (**A**) an FDNIRS-DCS study measurement and (**B**) a baseline neurodevelopmental outcome assessment with a patient.

During a patient’s initial hospital stay at CCHU, we aim to conduct daily measurements, excluding immediately following surgery. FDNIRS-DCS measurements are safe, fast (30 s per location), and do not perturb the infant. The optical device utilized (MetaOx, ISS Inc., Champaign, IL, USA) is described elsewhere [28]. For each measurement, a hospital staff member escorts a parent and patient to the designated exam room. Infants are measured while asleep or awake in their caregiver’s lap (Figure 2A). The optical probe is gently placed on the infant’s scalp in up to six different locations (F3, F4, F7, F8, T7, and T8), covering bilateral frontal and temporal areas defined by the conventional EEG 10-10 system (Supplementary Figure S1). Additionally, a 3D Polhemus Fastrak position-tracking sensor (Polhemus, VT, USA) has been incorporated in the FDNIRS-DCS probe to co-register optical measurements with CT images. This is achieved using radiographic markers placed on anatomical landmarks (nasion, left and right auricular points) on each subject’s head prior to clinical imaging. 3D-tracking is performed during the FDNIRS-DCS measurements to enable accurate registration between optics and imaging, depict underlying anatomical structure, and ensure adequate brain interrogation of optical signals.

During the FDNIRS-DCS measurement period, physiological parameters such as heart rate, blood pressure, and arterial saturation (SaO_2_) are recorded from a bedside patient monitor (Philips IntelliVue MP30, Philips Healthcare, Cambridge, MA, USA). In addition to periodic non-invasive blood pressure recordings using a standard clinical cuff, a continuous pulsatile arterial blood pressure (ABP) waveform is acquired non-invasively using a Finapres device (Finometer, Finapres Medical Systems, Amsterdam, The Netherlands) throughout the FDNIRS-DCS recording. We follow the method validated by Yiallourou et al. [29,30,31] for utilizing the Finapres with infants. For each measurement, an appropriate-size Finometer cuff (small, medium, or large) is selected and placed around the infant’s wrist. Measurements using this system are performed cyclically with periods of rest to prevent pooling of venous blood in the hand. In addition, hemoglobin in the blood (HgB), which is documented in each patient’s clinical chart, is collected for calculation of CMRO_2_.

All measurements are scheduled to minimize the likelihood of the participants being in an agitated state (e.g., shortly after feeding or immediately after clinical procedures). The total measurement time, including setup, is less than 60 min. All equipment coming into contact with the patient or parent is sanitized thoroughly following the measurement period.

#### 3.1.4. Neurodevelopmental Outcome Assessment

On admission and prior to surgery, a baseline Bayley Scales of Infant and Toddler Development (BSID-3) and Vineland Adaptive Behavior Scales (Vineland-3) is administered upon an enrolled participant by a trained researcher (Figure 2B). The BSID-3 is a play-based assessment of cognitive, language, and motor development, and the Vineland-3 is a parent/caregiver self-report questionnaire completed through an interview conducted by trained study staff [32].

At the six and 12 month post-operative and 24-month old visits, the neurodevelopmental outcome assessment is repeated and the results will be entered into a research database for analysis and correlation with other assessment measures. We have previously used a culturally modified version of the BSID successfully in Ugandan infants [33], similar to previous reports by Drotar et al. [34]. Due to the fact that language complexity and cultural influence may become confounding factors past 18 months of age, only the Cognitive and Motor Scales will be administered at the 24 months of age review. We currently do not have a validated instrument for language assessment at 24 months in any local languages.

### 3.2. Data Security, Storage, and Sharing

The Study ID Tracking Log, which lists the medical record numbers corresponding to the patient identification codes and other necessary protected health information (PHI) acquired for research purposes, is stored electronically in a separate secure file. Patients’ clinical (diagnostic tests, imaging, etc.) and measurement information (date of measurement, measurement location, which instruments used, bedside notes, etc.) are stored in REDCap and other secure electronic databases. Only members of the study team who are involved in analysis and have completed both Health Insurance Portability and Accountability Act (HIPAA) and Collaborative Institutional Training Initiative (CITI) training for human research have access to the clinical history of all enrolled infants. Any regulatory documentation that must be stored on paper is kept in a binder on site and does not contain identifying information. The portions of the regulatory binders maintained on paper, as well as the signed consent forms, are filed in the research record and stored in a locked cabinet in a locked room at CCHU.

Data collected at CCHU is shared primarily between the listed study collaborators upon request. A Data Usage Agreement (DUA) has been instituted to this effect. The data is used exclusively for research purposes.

### 3.3. Data Analysis

#### 3.3.1. Data Analysis Platform

We developed a custom cloud-based platform for automated transfer and analysis of study data and to connect collaborators between all sites. A key goal of our approach is to also provide rapid feedback to the operators of the study device at CCHU to ensure the quality of the research data. In our previous studies conducted at BCH, FDNIRS-DCS data was collected under supervision of spectroscopy experts and data was assessed later in post-processing. Here, we aim to provide this analysis online and in real-time so on-site operators have immediate feedback on data quality.

This pipeline will facilitate data transfer from the research device and processing using a hybrid of local and cloud computing. Data is streamed from the device and processed using custom FDNIRS-DCS analysis modules written in python, and executed by Apache Spark Streaming, to convert raw spectroscopy data into hemodynamic variables. Raw data and processed results are automatically written to three endpoints using Spark: (1) Raw data are archived in a cloud-based distributed file system for long-term retention; (2) Processed results are transferred to a cluster for future big-data analysis; (3) Processed results are also written to a database for display in a live dashboard through dedicated webservers. The dashboards provide device operators with measurement feedback and enable researchers at all sites to track study progress using the same, up-to-date metrics. In addition, custom post-processing is facilitated through interactive python and R notebooks on dedicated JupyterHub servers. A minimal subset of these web services and initial data processing will be replicated locally at CCHU to ensure on-site operators receive feedback regardless of the hospital’s internet connection. Following any outages, cloud resources seamlessly integrate when the connection is re-established.

#### 3.3.2. Cerebral Hemoglobin Concentrations, Calculations, and Oxygen Metabolism Estimation

Using FDNIRS, concentrations of oxygenated (HbO) and deoxygenated hemoglobin (HbR) can be determined by fitting the measured absorption coefficient at each wavelength with the hemoglobin extinction coefficients reported in literature [35]. From these measures, total hemoglobin concentration (HbT = HbO + HbR) and SO_2_ (SO_2_ = HbO/HbT) are calculated. For DCS measures, an index of cerebral blood flow (CBF_i_) is estimated by fitting the normalized temporal autocorrelation (g2) function to a solution of the diffusion correlation equation [36,37]. Although CBF_i_ is expressed in units of mm^2^/s, rather than traditional perfusion units of mL/100 g/min, CBF_i_ correlates well with cerebral blood flow assessed by “gold standard” methods [38,39,40].

An index of CMRO_2_ (CMRO_2i_) in units Mol/dl × mm^2^/s is calculated as described previously [41] by combining optically measured SO_2_ and CBF_i_, SaO_2_ from a pulse-oximeter, and HgB from the complete blood count (CBC) test performed upon admission (CMRO_2_ = HgB × CBF × (SaO_2_ − SO_2_)).

#### 3.3.3. CT and US Imaging Analysis

To calculate brain and CSF volumes for CT images we use an established automation software tool that implements a particle filter image segmentation algorithm [42]. This approach has been previously reported in hydrocephalus infants [17]. Volumes of extra-axial and subdural CSF are also quantified when observed. To determine each patient’s normative growth percentile, brain volumes are converted to Z-scores based on previously described age- and sex-adjusted volume distributions derived from normal pediatric brain imaging archived at the NIH Pediatric MRI Data Repository at the Montreal Neurological Institute [43].

HUS imaging will be analyzed manually to characterize ventricle size through measuring the anterior horn width (AHW) and ventricular index (VI). Transcranial doppler measurements of systolic and diastolic flow velocities (FV_s_ and FV_d_) are estimated from the flow waveforms of the anterior and middle cerebral arteries. Pulsatility index (PI), a value reflecting cerebrovascular resistance and intracranial pressure, can be calculated as (FV_s_ − FV_d_)/FV_mean_. In addition, 3D images are achieved by reconstructing successive 2D images acquired over a 60° arc using a motorized housing unit to translate the transducer at a constant speed. Custom software developed by the Fenster Lab at Western University allows for manual segmentation of ventricles and estimation of CSF volume [44]; previous work has demonstrated its capabilities in infants with PHH [45]. Segmentation of the ventricles is performed for sagittal and coronal scans, and reconstructed to form a 3D-image of the ventricles.

### 3.4. Power Analysis and Statistical Plan

Assuming a 20% mortality rate at two years of age and a 98% follow-up rate, we anticipate 235 infants will be included in our analysis. Based on Fisher’s transformation of the Pearson correlation coefficient, we will have 80% power to detect a true correlation of 0.18 or greater between FDNIRS-DCS data at the time of the pre-operative measurement, which, for some patients could be a measurement at birth, and our primary outcome of cognitive score on the neurodevelopmental exam at 24 months of age.

To investigate the ability of post-operative changes in FDNIRS-DCS parameters to predict the likelihood of ETV/CPC failure within six months, we anticipate requiring sample sizes of 72 infants in the ETV failure group and 168 infants in the ETV survival group, achieving 80% power to detect a 10% difference in the area under an ROC curve (AUC) according to the null hypothesis of 80% and an AUC under the alternative hypothesis of 90% using a two-sided z-test at a significance level of 0.05.

Optical measurements, cerebral volumes from imaging, and neurocognitive scores from BSID-3 and Vineland-3 tests will be analyzed to elucidate the relationship between measured parameters and brain development/treatment outcome. Any association between optical metrics (CBF_i_, CMRO_2i_) and brain growth will be determined using mixed-model regression analysis, with the former as the primary predictor of interest and Z-score for brain volumes as the response. Important covariates will include treatment failure requiring repeat surgery and confounders such as ETV surgery score [46], number of infections, number of operations, days spent in hospital, age at hydrocephalus incidence and treatment, and gender. We expect Z-scores for brain volume to be positively correlated with changes in CMRO_2_. However, since growth rates change rapidly in the first few months of life, regression coefficients are suspected to differ between baseline to six-month, six- to 12-month, and 12- to 24-month periods. We will, therefore, fit separate models for each interval. The same regression analysis will be performed using BSID-3 and Vineland-3 scores (whole and subdomains) as the response in order to determine the association between FDNIRS-DCS measures (SO_2_, CBF_i_, and CMRO_2i_) and neurodevelopmental outcome. Similarly, we hypothesize that children with greater increases in CMRO_2_ will achieve better scores in neurodevelopmental testing.

### 3.5. Staff Training

All research staff and co-investigators are required to read the protocol before assuming active roles in the project. Research staff in contact with subjects have undergone training in proper conduct, hygiene, and sanitization procedures. This includes both training specific to the study and training required by the participating hospital. Research staff responsible for conducting measurements using FDNIRS-DCS and other devices were asked to complete (1) an online training module on laser safety; (2) a virtual training course produced by the BCH team detailing the basic science behind optical monitoring, FDNIRS-DCS device setup, and measurement protocol; and (3) in-person training with a step-by-step evaluation of how to conduct a measurement. In-person and/or virtual refresher training will occur periodically for CCHU staff responsible for the FDNIRS-DCS measurements and the neurodevelopmental outcome assessments.

### 3.6. Data Quality Assurance

To ensure the sustained integrity of all acquired data for the duration of the study and mitigate sources of potential error, quality assurance checks are routinely conducted on all devices by CCHU staff. The MetaOx device undergoes a weekly stability test consisting of several measurements performed on two calibration blocks with known absorption and scattering properties to ensure accurate quantification of measured optical signals. In addition, the condition of the FDNIRS-DCS probe is assessed through a visual examination of the probe face and by monitoring the DCS laser output with a power measurement. To provide operator performance feedback to the CCHU team, we have utilized the online data dashboard described in Section 3.3.1 to discuss analyzed results soon after acquisition. Through regular data quality assessment, we are able to provide immediate feedback to maintain high data quality. Weekly quality assurance tests are also completed on the 3D-US and CT scanners to monitor their stability over time. Assessment of the 3D-US is accomplished through a weekly scan of an imaging phantom of known geometry. CT images are calibrated through biweekly scans of a CT imaging phantom consisting of materials with a known radiodensity.

Internal auditing will occur periodically throughout the course of the study to assure data completeness and accuracy. Auditing initiated by the CCHU Research Ethics Committee (CCHU-REC) or Uganda National Council of Science and Technology (UNCST) will occur annually upon request.

### 3.7. Reporting

Enrollment reports will be submitted to the sponsor (NIH), BCH IRB, and CURE Children’s Hospital of Uganda Research Ethics Committee (CCHU-REC) at the annual Continuing Review (CR). Minor deviations and exceptions are recorded in the Deviation Log to be submitted at the annual CR. In the unlikely scenario that an adverse event occurs during any part of this study, the event will be immediately reported to the PI, the CCHU site-PI, and IRB committees at CCHU and BCH. Adverse events temporally occurring due to participation in this study will be documented to note whether they are considered related to research activities.

### 3.8. Ethics Approval, Registration, and Dissemination

This study is approved and regulated by CCHU-REC, Uganda National Council of Science and Technology (UNCST), and Boston Children’s Hospital Institutional Review Board (BCHIRB). The study is also registered in ClinicalTrials.gov (registration number NCT03650101), Boston Children’s Hospital Institutional Review Board (IRB-P00029806), CURE Hospital Research Ethics Committee (CCHU-REC/01/019), and Uganda National Council of Science and Technology (UNCST HS 2710). Results of this study will be shared with the scientific community through the internet, conference presentations, and publications in peer-reviewed journals.

### 3.9. Study Status

This study is currently approved and actively enrolling participants. The current approved protocol is version 1.3 (version date August 2020). The first subject was consented in May of 2021. The BCH, CCHU, PSU, and WU teams hold virtual meetings several times each week to discuss study updates, preliminary results, management of data, device maintenance, and areas for study improvement. In addition to frequent virtual meetings, members of the BCH team will continue traveling to CCHU at least twice a year to ensure the study’s progress.

## 4. Discussion

Low-resource settings, such as sub-Saharan Africa, have a high prevalence of infant hydrocephalus, limited access to neurosurgical care, and are in dire need of sustainable treatment. At CCHU, the ETV/CPC procedure has replaced VPS placement as standard practice for infantile hydrocephalus; however, the prevalence and physiological basis of its failure warrant further exploration. FDNIRS-DCS has great potential to provide safe and convenient prognostic monitoring for hydrocephalus treatment in LMICs. This multi-site, international investigation seeks to understand the relationship between physiological measures, brain development, and treatment outcome in a low-resource, high-volume setting. This study is unique due to its complexity, involving multiple imaging modalities and a diverse research and clinical team. This translational study requires considerable effort for staff training, device setup, and data management. With these intricacies in mind, we anticipate several challenges, including but not limited to device maintenance, data transfer/management, and logistical complications related to the ongoing COVID-19 pandemic such as travel.

The coordination of patient follow-ups, including tracking, scheduling, and patient transportation, is the responsibility of the CCHU Tracking Coordinator. Previous studies at CCHU have shown high patient retention (99%) following the first successful follow-up visit [16]. A high patient retention rate is maintained through the emphasis placed on the value of these visits at enrollment and discharge after initial inpatient stay, periodic check-in calls between visits with a focus on both family and community engagement, as well as continued information on post-operative care. Two weeks prior to each follow-up, the Tracking Coordinator will contact the caregiver or a member of the Village Health Team (a community-based committee managing healthcare for local residents). If contact is limited, the Tracking Coordinator will make a home visit. If long-distance travel is required, overnight accommodations at the hospital’s hostel will be granted. If there is no contact from the primary or secondary contacts after 2–3 attempts made by the Tracking Coordinator, and the home visit proves unsuccessful in locating the patient, the visit is logged as a loss to follow-up.

We acknowledge the impact that the COVID-19 pandemic has and may continue to have on the expected number of eligible infants admitted to CCHU as well as the number of on-site staff members available carry out all study procedures. In the past year, Uganda has experienced multiple lockdowns to mitigate the spread of the virus, which have imposed travel restrictions (i.e., a decrease in accessibility to inter-district travel methods and lowered hospital staff capacity levels). Such travel restrictions pose a significant barrier to families who have greater distances to travel to reach CCHU, while limited CCHU staff capacity, and in turn limited research staff capacity, may impede the ability of the research staff to recruit every eligible patient and successfully complete all study components for those already enrolled. In addition to hospital patient and staff capacity constraints and the imposed travel restrictions due to the COVID-19 pandemic, parental fear that themselves or their child will contract the virus by coming into the hospital is a factor that has an effect on recruitment rate, as there are fewer eligible infants admitted. Research staff have previously reported the apprehension felt by parents and observed a subsequent decrease in hospital admission rates. In order to mitigate the spread of COVID-19, all CCHU research staff wear the necessary PPE, maintain social distancing at times when the study does not require contact, and uphold proper sanitization procedures of commonly touched surfaces. In the event that a CCHU staff member is exposed to an individual who tests positive for COVID-19, they are required to follow the guidelines delineated in the CCHU COVID-19 Risk Management Protocol.

## Data Availability

Data is contained within the article or Supplementary Materials. The data presented in this study are available upon request.

## Supplementary Materials

The following supporting information can be downloaded at: https://www.mdpi.com/article/10.3390/metabo12010078/s1, Figure S1: Illustration of the 10-10 EEG electrode system. FDNIRS-DCS locations are outlined in red.
