# Supplementary figures and images for: Improving Infant Hydrocephalus Outcomes in Uganda: A Longitudinal Prospective Study Protocol for Predicting Developmental Outcomes and Identifying Patients at Risk for Early Treatment Failure after ETV/CPC
Source: Metabolites. 2022 Jan 14;12(1):78. doi: 10.3390/metabo12010078 (PMC8781620; doi:10.3390/metabo12010078)

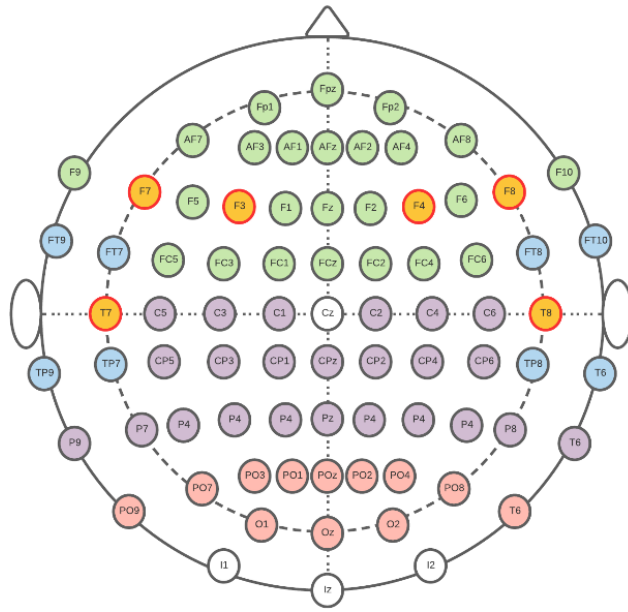

**Figure S1.** Illustration of the 10-10 EEG electrode system. FDNIRS-DCS locations are outlined in red.

Supplement: Supplementary file 1 [file metabolites-12-00078-s001.zip › metabolites-1530826-supplementary.pdf]
